# A Smoothened receptor agonist is neuroprotective and promotes regeneration after ischemic brain injury

**DOI:** 10.1038/cddis.2014.446

**Published:** 2014-10-23

**Authors:** O V Chechneva, F Mayrhofer, D J Daugherty, R G Krishnamurty, P Bannerman, D E Pleasure, W Deng

**Affiliations:** 1 Department of Biochemistry and Molecular Medicine, School of Medicine, University of California, Davis, CA 95817, USA; 2 Institute for Pediatric Regenerative Medicine, Shriners Hospitals for Children, 2425 Stockton Boulevard, Sacramento, CA 95817, USA; 3 Medical College, Hubei University of Arts and Science, Xiangyang, Hubei 441053, China

## Abstract

Ischemic stroke occurs as a result of blood supply interruption to the brain causing tissue degeneration, patient disabilities or death. Currently, treatment of ischemic stroke is limited to thrombolytic therapy with a narrow time window of administration. The sonic hedgehog (Shh) signaling pathway has a fundamental role in the central nervous system development, but its impact on neural cell survival and tissue regeneration/repair after ischemic stroke has not been well investigated. Here we report the neuroprotective properties of a small-molecule agonist of the Shh co-receptor Smoothened, purmorphamine (PUR), in the middle cerebral artery occlusion model of ischemic stroke. We found that intravenous administration of PUR at 6 h after injury was neuroprotective and restored neurological deficit after stroke. PUR promoted a transient upregulation of tissue-type plasminogen activator in injured neurons, which was associated with a reduction of apoptotic cell death in the ischemic cortex. We also observed a decrease in blood–brain barrier permeability after PUR treatment. At 14 d postinjury, attenuation of inflammation and reactive astrogliosis was found in PUR-treated animals. PUR increased the number of newly generated neurons in the peri-infarct and infarct area and promoted neovascularization in the ischemic zone. Notably, PUR treatment did not significantly alter the ischemia-induced level of Gli1, a Shh target gene of tumorigenic potential. Thus our study reports a novel pharmacological approach for postischemic treatment using a small-molecule Shh agonist, providing new insights into hedgehog signaling-mediated mechanisms of neuroprotection and regeneration after stroke.

Stroke is the third leading cause of death and the main cause of disabilities in industrial countries. Thrombolytic therapy with tissue-type plasminogen activator (tPA) is the only FDA-approved treatment of stroke. tPA clinical use is limited to a narrow time window of safe administration and associated with dangers of intracranial hemorrhage.^1^ Neuroprotective compounds, safe and effective when administered at later time points after insult, remain to be identified.

The sonic hedgehog (Shh) signaling has an essential role in central nervous system (CNS) development,^2,3^ regulating the generation and survival of neurons and oligodendrocytes.^4, 5, 6^ In the adult CNS, Shh is expressed in forebrain neurons and astrocytes, cerebellar Purkinje and motor neurons.^3^ Shh controls proliferation of neuronal progenitors and reactivity of astrocytes.^7, 8, 9, 10^ Perivascular astrocytes express Shh to maintain blood–brain barrier (BBB) integrity.^11^

Shh is a morphogenic protein that binds to its 12-pass transmembrane receptor Patched (Patched 1 (Ptch1) in mammals) localized to the primary cilium in vertebrates. Ptch1 activation releases the inhibition of G protein-coupled receptor Smoothened (SMO), allowing downstream nuclear translocation of Gli transcription factors, key regulators of the Shh pathway.^2^ Non-canonical Shh signaling, lacking Gli-mediated transcription, has also been reported.^12^

Shh signaling is activated in response to CNS injury, and the time window and the cellular players involved is dependent on the type and severity of insult. Multiple studies showed the beneficial role of Shh signaling in various models of neurological conditions, including stroke,^10,13, 14, 15, 16, 17, 18^ acute brain injury,^19^ Parkinson's disease,^20,21^ Alzheimer's disease,^22^ multiple sclerosis and demyelination,^11,23,24^ spinal cord injury,^25,26^ amyotrophic lateral sclerosis,^27^ and glucocorticoid-induced cerebellar injury in neonates.^28^ However, the potential of pharmacological activation of Shh signaling in injured CNS and associated neuroprotective mechanisms are unknown.

Purmorphamine (PUR) is a purine-derivative small-molecule agonist of SMO receptors.^29^ PUR was introduced by Schultz and co-workers as an osteogenic compound for differentiation of mouse mesenchymal progenitors into osteoblasts.^30^ Using an *in vitro* amyotrophic lateral sclerosis model, Peterson and Turnbull^27^ showed that PUR protected hippocampal neurons against oxidative challenge. A barrier-promoting effect of PUR was reported in BBB–endothelial cell culture model.^11^ To date, the therapeutic potential of PUR in ischemic stroke has not been examined.

Here we investigated the neuroprotective and regenerative properties of PUR in an experimental model of ischemic stroke. We also characterized the ischemia-induced activity of endogenous Shh signaling in the brain.

## Results

### PUR protects cortical tissue against ischemic injury

**Figure 1:**
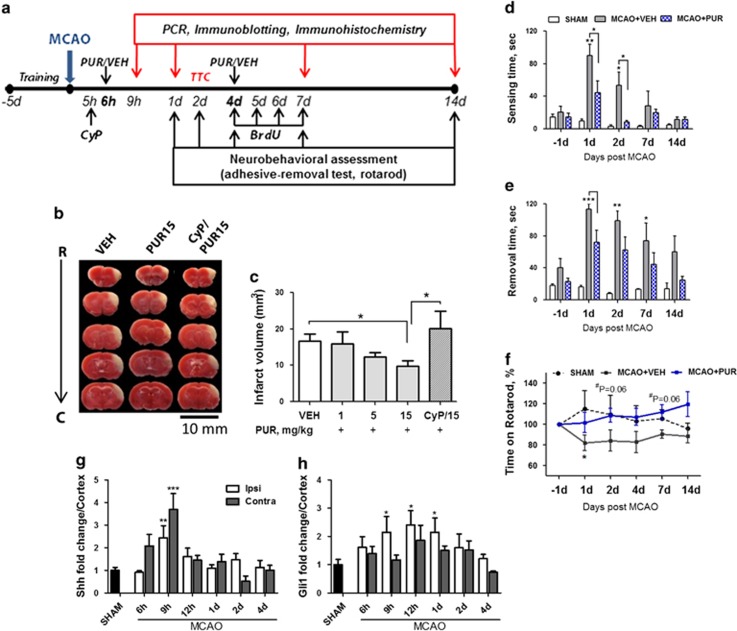
PUR is neuroprotective in a mouse model of ischemic stroke. (**a**) Experimental design. PUR or VEH was administered i.v. at 6 h and 4 d after MCAO. Shh antagonist CyP (10 mg/kg) was injected i.v. 40 min before PUR administration. (**b**) Infarct area detected by TTC staining in animals treated with VEH, PUR (15 mg/kg) or CyP and PUR (15 mg/kg). (**c**) Quantitative analysis of TTC (*n*=8–10). Data are the means±S.E.M.; *P<0.05. Time to (**d**) sense and (**e**) remove adhesive from the contralateral front pawn in the adhesive-removal test (*n*=8–9). (**f**) Time on rotarod shown as the percentage to the time recorded on the last day of training (*n*=8–10; *P<0.05 compared with SHAM; ^#^VEH *versus* PUR). mRNA levels of (**g**) Shh and (**h**) Gli1 in the cortex after MCAO (*n*=3–6; *P<0.05; **P<0.01, ***P<0.001)

The therapeutic time window of neuroprotective compounds is critical in defining the success of stroke therapy. Dellovade *et al.*^15^ described the ability of an agent of the hedgehog pathway to reduce infarct volume and neurological deficits when administered intravenously (i.v.) at 6 h after stroke. We followed a similar poststroke treatment paradigm to examine the neuroprotective activity of PUR. At 6 h after permanent middle cerebral artery occlusion (MCAO), animals were injected i.v. with 1, 5 or 15 mg/kg of PUR or vehicle (VEH) (Figure 1a). The infarct area was examined 2 days later using 2,3,5-triphenyltetrazolium chloride (TTC). A 40% reduction of infarct volume was found in animals treated with PUR at 15 mg/kg compared with VEH (Figures 1b and c). A dose of 15 mg/kg of PUR was used throughout the study. Neuroprotective effect of PUR was abolished by preadministration of SMO receptor antagonist cyclopamine (CyP; Figures 1b and c). CyP alone did not affect the infarct volume when tested in a pre- and post-MCAO treatment paradigm (Supplementary Figure S1A). Intraperitoneal (i.p.) administration of PUR at 6 h after stroke did not show neuroprotective activity (Supplementary Figure S1B), presumably due to specific pharmacokinetic properties of the compound.^26^

Adhesive-removal and rotarod behavioral tests revealed improved neurological outcome after cerebral ischemia in PUR-treated animals when compared with VEH. PUR decreased the time required to sense (Figure 1d) and remove (Figure 1e) adhesive 1 d after stroke and further improvement in sensing was observed on day 2. No significant differences were detected later (7 and 14 d post-MCAO) due to improved performance in both groups. On the rotarod, PUR-treated animals demonstrated enhanced motor function at 2 d postinjury compared with VEH (Figure 1f). Differences were still apparent at 4, 7 and 14 d post-MCAO. These findings demonstrated that a single dose i.v. administration of PUR at 6 h after stroke was neuroprotective.

### Shh signaling is upregulated in the cortex early after stroke

**Figure 2:**
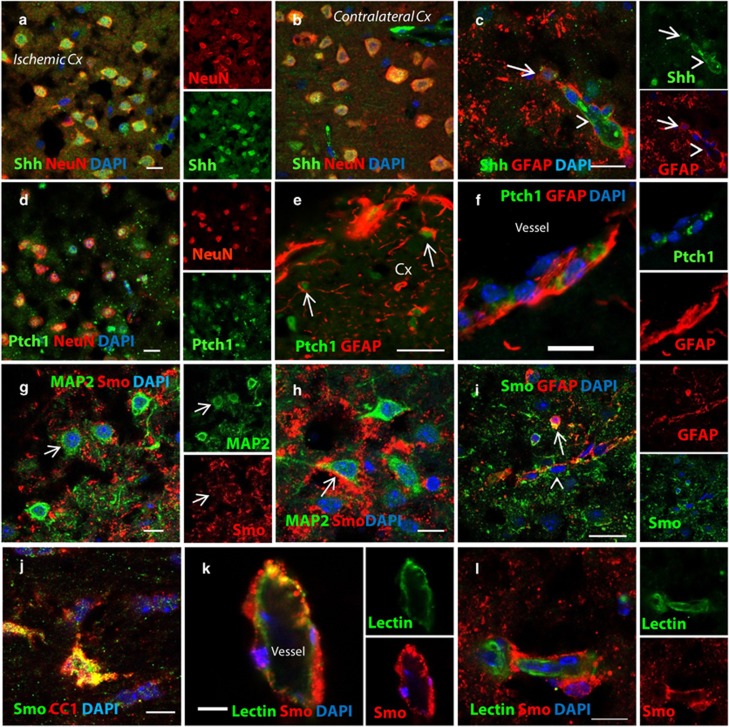
Shh signaling is upregulated in the ischemic cortex at 9 h after MCAO. Neurons (**a**) in the ischemic area and (**b**) in the contralateral cortex express Shh. (**c**) Some GFAP+ astrocytes (arrow) and endothelial cells (arrow head) express Shh at 9 h after stroke. (**d**) Neurons in the infarct area express Shh receptor Ptch1. Ptch1+ astrocytes present in the (**e**) ischemic parenchyma and (**f**) perivascular units. (**g** and **h**) Neurons in the ischemic area express Shh co-receptor Smo. (**i**) Smo+ parenchymal (arrow) and perivascular (arrow head) astrocytes and (**j**) oligodendrocytes in the ischemic cortex. Vascular endothelial cells expressing (**k**) Smo and (**l**) Smo-negative endothelial cells in the ischemic area. Scale bar, 50 *μ*m for **e** and **i**; 20 *μ*m for (**c**, **d**, **f**–**h**, **k** and **l**) 10 *μ*m for (**a**, **b** and **j**). Cx, cortex

To investigate the mechanism of PUR-induced neuroprotection, we first characterized endogenous Shh signaling after MCAO. We found that Shh mRNA was transiently upregulated in ipsilateral and contralateral cortex at 9 h and returned to control level by 12 h after injury (Figure 1g). At 9 h after stroke, 90% of Shh+ cells in the ipsilateral and contralateral cortex co-expressed neuronal marker NeuN (Figures 2a and b,Supplementary Figure S1C). Rare Shh+ astrocytes (Figure 2c, arrow) and endothelial cells surrounded by perivascular astrocytes (Figure 2c, arrow head) were detected in the ischemic cortex. Shh receptor Ptch1 was predominantly expressed in neurons in the ischemic cortex (Figure 2d) and occasional Ptch1+ neurons were found on the contralateral side (Supplementary Figure S1D). Some parenchymal (Figure 2e, arrow) and perivascular (Figure 2f) astrocytes in the infarct area and on the contralateral side expressed Ptch1. Ptch1+ endothelial cells (Supplementary Figures S1E and F) and microglia (Supplementary Figure S1G) were detected in the ipsilateral cortex.

**Figure 3:**
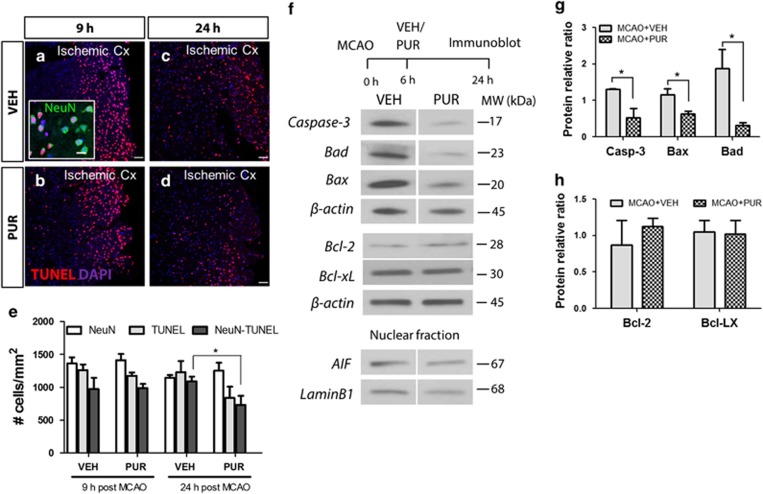
Effect of PUR on Shh signaling and inflammation. Level of mRNA coding for (**a**) Shh, (**b**) Ptch1, (**c**) Smo and (**d**) Gli2 in SHAM- (*n*=6), VEH- (*n*=11) and PUR-treated animals (*n*=11) at 9 h after MCAO. (**e**) Gli1 mRNA level at 9 and 24 h (*n*=6) after stroke. (**f**) Immunoblot analysis of Gli1 and Gli2 in the nuclear protein fraction isolated from ischemic cortex at 9 h after stroke (*n*=4–5). (**g**) Quantification of protein level normalized to loading control Lamin B1 (*n*=5). Bands cropped from the same blot are separated by a vertical line. mRNA expression level of pro-inflammatory cytokines (**h**) IL6, (**i**) IL1*β* and (**j**) TNF*α* detected at 9 (*n*=11) and 24 h (*n*=6) after MCAO in VEH- and PUR-treated animals. Data are the means±S.E.M.; *P<0.05; **P<0.01; ***P<0.001

At 9 h after injury, Shh co-receptor SMO was predominantly expressed not only in neurons (Figures 2g and h) but also in perivascular and parenchymal astrocytes (Figure 2i), CC1+ oligodendrocytes (Figure 2j) and vascular endothelial cells in the ischemic cortex (Figure 2k). SMO-negative endothelial cells were also present in the ischemic cortex (Figure 2l). Gli1 mRNA was elevated in the ischemic area at 9, 12 and 24 h postinjury (Figure 1h). Gli1 protein level of the nuclear fraction was increased at 9 h after stroke (Figures 3f and g).

### PUR inhibits apoptosis induced by ischemic stroke

**Figure 4:**
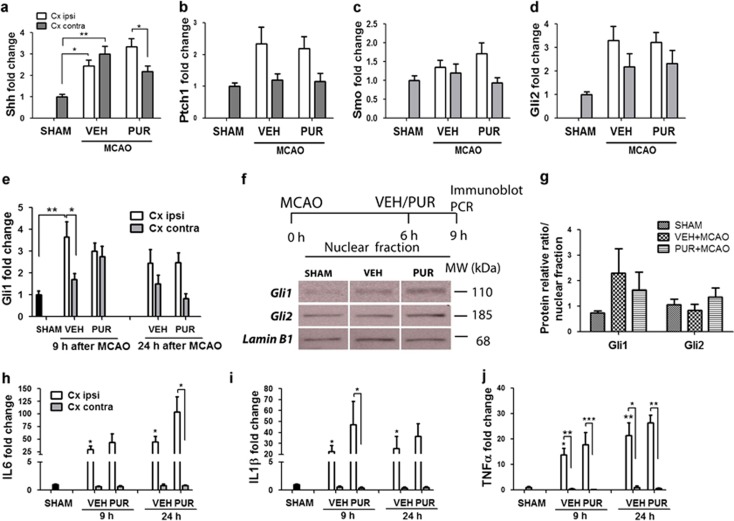
PUR reduces apoptotic cell death in the ischemic cortex after MCAO. Apoptotic cells in the ischemic cortex of (**a** and **c**) VEH- and (**b** and **d**) PUR-treated animals at 9 and 24 h after MCAO (representative image of *n*=4–5; scale bar, 50 *μ*m). Apoptotic neurons (insert in panel (**a**); scale bar, 20 *μ*m). Cx, cortex. (**e**) Quantitative analysis of apoptotic cells in the ischemic cortex (*n*=4–5). Data are the means±S.E.M.; *P<0.05. (**f**) Representative blots for cleaved caspase-3, Bad, Bax, Bcl-2 and Bcl-xL in the cytoplasmic and AIF in the nuclear protein fraction. Bands cropped from the same blot are separated by a vertical line. (**g** and **h**) Quantification of protein levels relative to loading control *β*-actin. Data are the means±S.E.M.; *n*=6; *P<0.05

Cells in the ischemic core degenerate shortly after stroke,^31^ and at 6 h postinjury significant damage to brain tissue is detectable.^32^ However, cells in the peri-infarct penumbra undergo delayed apoptotic cell death and can be rescued by neuroprotective treatment. We found a high number of TUNEL+ cells in the ischemic cortex of PUR- and VEH-treated animals at 9 h after stroke (3 h after treatment) (Figures 4a and b). The majority of apoptotic cells expressed NeuN (Figures 4a and e, insert). At 24 h (18 h after treatment), a reduction of apoptotic cell numbers was observed in PUR-treated animals (Figures 4c–e). Cresyl violet staining confirmed that this decrease was not due to cell death as no noticeable change in cell density in the ischemic area was observed between VEH- and PUR-treated groups at this time point (Supplementary Figures S1H and J). A significant reduction in the protein level of cleaved caspase-3 and pro-apoptotic mediators Bad and Bax were detected in PUR-treated animals at 24 h (Figures 4f and g). No differences in the expression level of pro-survival factors Bcl-2 and Bcl-xL or apoptosis-inducing factor were observed (Figures 4f and h). Thus our data showed that late neuroprotection by PUR was associated with inhibition of apoptosis.

### PUR does not alter the stroke-induced level of Shh signaling or inflammation

We next evaluated the involvement of Shh signaling molecules in the anti-apoptotic activity of PUR by examining their expression in the ischemic cortex at 3 h after treatment (9 h postinjury). No differences in the mRNA level of Shh, Ptch1, SMO, Gli2 or Gli1 (Figures 3a–e) were found between VEH and PUR-treated groups. A reduction of Shh mRNA, however, was observed at 9 h after stroke in the contralateral cortex of PUR-treated animals (Figure 3a). No differences in the Gli1 or Gli2 protein level between VEH- and PUR-treated animals were detected (Figures 3f and g).

Inflammation triggers the activation of Shh signaling after brain injury or in cancer cells.^19,33^ We found that mRNA levels of pro-inflammatory cytokines IL6, IL1*β* and TNF-*α* were elevated in the ischemic cortex at 9 and 24 h after stroke (Figures 3h–j). PUR did not affect the ischemia-induced level of inflammatory mediators.

### Neurons in the ischemic cortex express tPA shortly after PUR treatment

**Figure 5:**
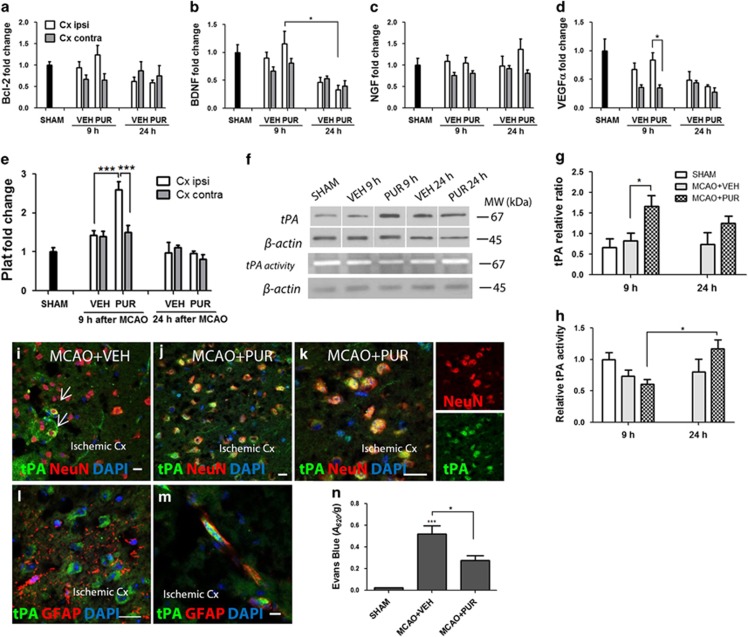
PUR increases the expression of tPA in ischemic neurons early after treatment. Levels of mRNA coding for (**a**) Bcl-2, (**b**) brain-derived neurotrophic factor (BDNF), (**c**) nerve growth factor (NGF), (**d**) vascular endothelial growth factor (VEGF) and (**e**) Plat at 9 (*n*=6–11) and 24 h (*n*=6) after MCAO. (**f**) Immunoblot analysis of tPA protein level and enzymatic activity after stroke. Quantification of (**g**) tPA protein level and (**h**) tPA activity normalized to *β*-actin (*n*=6). Bands cropped from the same blot are separated by a vertical line. Data are the means±S.E.M.; *P<0.05; **P<0.01; ***P<0.001. (**i**) tPA+ neurons in the ischemic cortex at 9 h after MCAO in VEH- (arrows) and (**j** and **k**) PUR-treated animals (representative images of *n*=4). (**l**) Parenchymal astrocytes in the ischemic cortex did not express tPA at 9 h after stroke (**m**) while some perivascular astrocytes were tPA+. Scale bar, 20 *μ*m for I-L; 10 *μ*m for M. Cx, cortex. (**n**) BBB permeability at 10 h after MCAO. Data are the means of *n*=5±S.E.M.; *P<0.05; ***P<0.001

To further investigate the underlying mechanism of PUR-induced neuroprotection, we examined the expression of several factors previously identified in association with Shh-mediated responses.^22,34^ No differences in the mRNA levels of pro-survival factor Bcl-2 (Figure 5a) and neurotrophic factors BDNF (Figure 5b), NGF (Figure 5c) or VEGF*α* (Figure 5d) were found between VEH- and PUR-treated animals at 9 or 24 h after stroke. However, at 9 h after MCAO, the mRNA level of Plat, encoding for tPA (Figure 5e), and the tPA protein level were dramatically upregulated in the ischemic cortex of PUR-treated animals (Figures 5f and g). At 24 h after injury, tPA mRNA and protein expression returned to control levels. Notably, tPA proteolytic activity was reduced in the ischemic cortex of VEH- and PUR-treated animals at 9 h after stroke (Figures 5f and h). By 24 h, tPA activity was significantly increased in PUR-treated animals. Early after stroke, tPA was dominantly expressed by neurons in the ischemic cortex (Figures 5i–k). No parenchymal but some perivascular astrocytes expressed tPA at 9 h after insult (Figures 5l and m).

Increased cerebrovascular permeability has been linked to tPA activity after ischemic stroke.^35^ We examined whether PUR-induced tPA expression affected BBB integrity. A significant uptake of Evans Blue (EB) dye was detected in the ipsilateral cortex of VEH-treated animals at 10 h after MCAO, indicating the breakdown of BBB. Notably, BBB permeability was reduced after PUR treatment (Figure 5n).

### PUR attenuates reactive astrogliosis and inflammation after stroke

**Figure 6:**
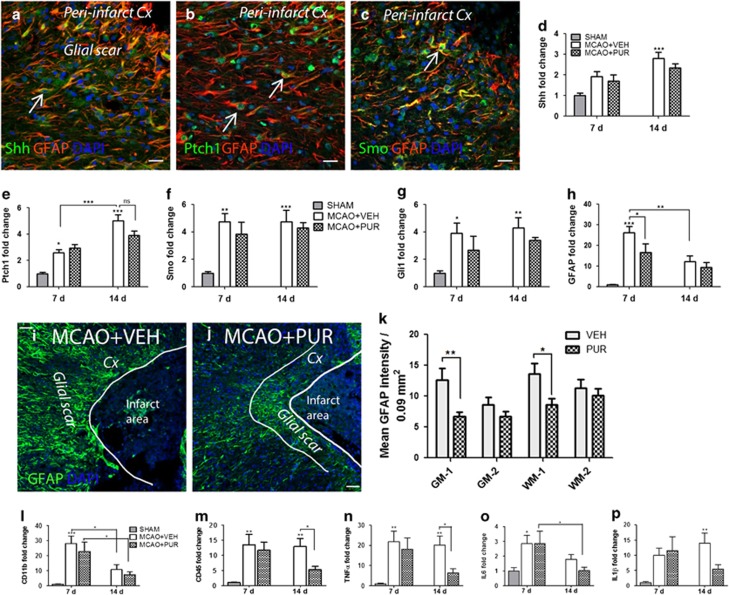
PUR reduces astrogliosis and inflammation at a later time point after stroke. Reactive astrocytes in the glial scar express (**a**) Shh, (**b**) Ptch1 and (**c**) Smo. Representative images of *n*=8–10. mRNA expression levels of (**d**) Shh, (**e**) Ptch1, (**f**) Smo, (**g**) Gli1 and (**h**) GFAP in the ischemic cortex at 7 and 14 d after stroke (*n*=6–7). Expression of GFAP in the glial scar at 14 d after MCAO in (**i**) VEH- and (**j**) PUR-treated animals. Representative images of *n*=8–10. (**k**) Mean GFAP intensity in the glial scar in areas corresponding to grey (GM1) or white (WM1) matter in the peri-infarct zone and areas located at 300 *μ*m from the border to the ischemic zone (GM2, WM2; *n*=8–10). Levels of mRNA coding for (**l**) CD11b, (**m**) CD45, (**n**) TNF*α*, (**o**) IL6 and (**p**) IL1*β* at 7 and 14 d after stroke (*n*=6–7). Data are the mean±S.E.M.; *P<0.05; **P<0.01; ***P<0.001. Scale bar, 50 *μ*m for **a**–**c**, **i** and **j**. Cx, cortex; NS, not significant

**Figure 7:**
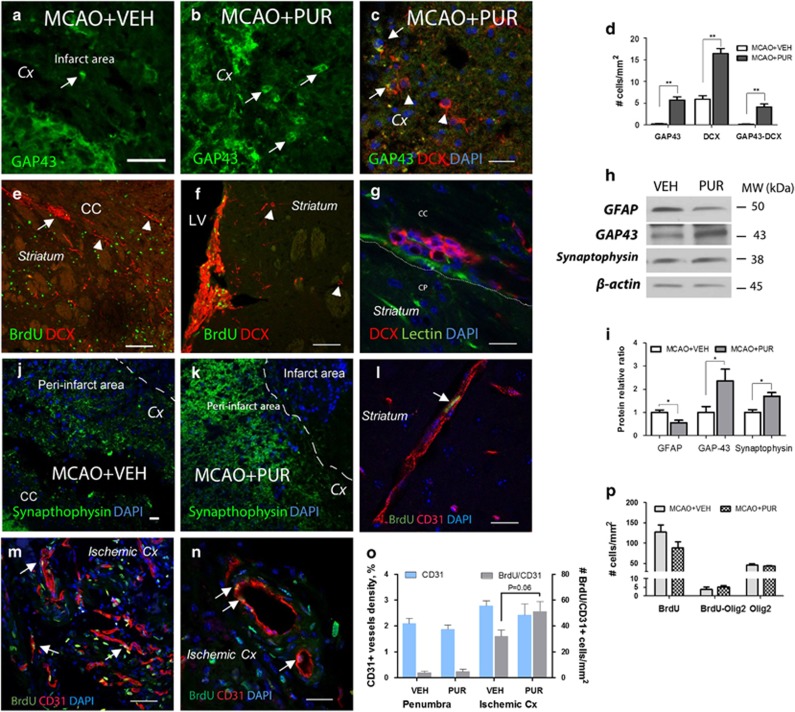
PUR promotes regeneration after stroke. Expression of GAP43 in the ischemic zone of (**a**) VEH- and (**b**) PUR-treated animals at 14 d after stroke. GAP43+ cells with neuroblast morphology (arrows). (**c**) DCX+ (arrow heads) and GAP43/DCX+ (arrows) cells in the ischemic cortex of PUR-treated animals. (**d**) Quantitative analysis of GAP43, DCX and GAP43/DCX+ cells. Cells were counted in eight 300 × 300 *μ*m^2^ squares in the ischemic area. Data are the mean±S.E.M. of *n*=8–10; **P<0.01. (**e**) DCX+ cells form clusters (arrow) and chains (arrowhead) of migrating neuroblasts in the CC. (**f**) DCX+ cells (arrowhead) found to detach and migrate from SVZ toward the ischemic area. (**g**) DCX+ cells migrate along blood vessels. (**h**) Immunoblot for GFAP, GAP43, synaptophysin and *β*-actin at 14 d after MCAO. Representative blots of *n*=4. (**i**) Quantification of protein levels relative to loading control *β*-actin. Data are the means of *n*=4±S.E.M.; *P<0.05. Expression of synaptophysin in the peri-infarct area of (**j**) VEH- and (**k**) PUR-treated animals. (**l**) Newly generated endothelial cells found in the penumbra (arrow). (**m**) Neovascularization in the infarct area. Bromodeoxyuridine (BrdU)/CD31 labels endothelial cells generated between 4 and 7 d after MCAO (arrows). (**n**) Newly generated endothelial cells as part of large size vessels in the infarct area. (**o**) Quantitative analysis of vascular density (CD31+ vessels) and newly generated (BrdU/CD31+) endothelial cells found in the penumbra and ischemic core at 14 d after stroke. Vascular density and endothelial cell numbers were analyzed in two 300 × 300 *μ*m^2^ squares placed in the peri-infarct or infarct cortex. Data are the means of *n*=5–7±S.E.M. (**p**) Quantitative analysis of BrdU+, Olig2+ and BrdU/Olig2+ cells. Cells were counted in three 300 × 300 *μ*m^2^ squares in the peri-infarct area of VEH- and PUR-treated animals. Data are the mean±S.E.M.; *n*=5–7. Scale bar, 50 *μ*m for (**a**–**c**, **e**, **f**, **j**, **k** and **m**) 20 *μ*m for (**g** and **l**) **N**. CP, caudate putamen; Cx, cortex; LV, lateral ventricle

Following an initial phase of stroke-induced degeneration and inflammation, regenerative mechanisms are initiated in the brain after MCAO.^32,36^ To promote regeneration, a second injection of PUR was administered at 4 d after insult (Figure 1a). Cortical tissue, including the ischemic core and peri-infarct region, was collected at 7 and 14 d after stroke for analysis of Shh signaling, inflammation and astrogliosis. Reactive astrocytes of the glial scar expressed Shh (Figure 6a), Ptch1 (Figure 6b) and SMO (Figure 6c) at 14 d after stroke. The mRNA levels of Shh (Figure 6d) and Ptch1 (Figure 6e) were upregulated in the ischemic cortex at 7 d with an additional increase at 14 d after injury. Smo (Figure 6f) and Gli1 (Figure 6g) mRNA levels were significantly elevated at 7 and 14 d. The mRNA level of glial fibrillary acidic protein (GFAP), a marker of reactive astrogliosis, was upregulated at 7 d after stroke (Figure 6h). Treatment with PUR did not change the mRNA levels of Shh, SMO or Gli1 (Figures 6d, f and g) but decreased Ptch1 mRNA at 14 d (Figure 6e). GFAP mRNA level was reduced at 7 d by PUR treatment (Figure 6h) and downregulated in both VEH- and PUR-treated groups at 14 d. Analysis of the glial scar tissue revealed a significant decrease of GFAP protein expression in PUR- *versus* VEH-treated mice at 14 d poststroke (Figures 6i–k,7h and i).

We further evaluated the effect of PUR on late stage inflammation and found that th mRNA levels of the microglia/macrophage marker CD11b (Figure 6l) and pro-inflammatory cytokine IL6 (Figure 6o) were increased in the ischemic cortex at 7 d but downregulated at 14 d after MCAO. Levels of mRNA coding for CD45 (Figure 6m), TNF*α* (Figure 6n) and IL1*β* (Figure 6p) were elevated at 7 and 14 d after stroke. Treatment with PUR decreased CD45, TNF*α* and IL1*β* mRNA levels at 14 d postinjury (Figures 6m, n and p). Thus PUR treatment at 6 h and 4 d after stroke resulted in reduction of reactive astrogliosis and late-stage inflammation.

### PUR promotes regeneration after stroke

To study regenerative properties of PUR, we examined the expression of neurite outgrowth protein GAP43,^37^ migrating neuroblasts marker doublecortin (DCX)^38^ and synaptophysin, a marker of synaptic plasticity,^39^ in the ischemic cortex at 14 d after stroke. We found GAP43+ cells with neuroblast morphology in the infarct area of VEH- and PUR-treated animals (Figures 7a and b, arrows). The majority of GAP43+ cells co-localized with neuroblast marker DCX (Figure 7c). Numbers of GAP43, GAP43/DCX and DCX+ cells were significantly higher in the ischemic area of PUR-treated animals compared with VEH (Figure 7d). DCX+ cell clusters and chains were located along the corpus callosum (CC; Figure 7e) and in the striatum (Figure 7f) of both VEH- and PUR-treated animals, suggesting their lateral migration from the subventricular zone (SVZ) toward the ischemic area. Migrating cells were present in close vicinity to blood vessels (Figure 7g). An elevated expression of synaptophysin, was detected in PUR-treated animals when compared with VEH at 14 d after stroke (Figures 7j and k). Western blotting confirmed increase of GAP43 and synaptophysin protein levels in the ischemic area of PUR-treated animals at 14 d after insult (Figures 7h and i).

Shh signaling is involved in vascular reconstruction after ischemic injury.^10^ We examined the effect of PUR on vascular density and neovascularization in the peri-infarct and infarct areas. Very rarely, vessels with newly formed (BrdU/CD31+) endothelial cells were found in the peri-infarct area of VEH- and PUR-treated animals (Figure 7l). In contrast, significant numbers of BrdU/CD31+ cells appearing as single cells or as part of large vessels were detected in the infarct area in both groups (Figures 7m and n). Quantification showed an increase of newly formed endothelial cells in the infarct area of PUR-treated animals when compared with VEH (Figure 7o). No difference in the density of CD31+ vessels was detected between VEH- and PUR-treated groups in the peri-infarct and infarct regions (Figure 7o).

Given the known role of Shh signaling in oligodendrogenesis,^19^ we next examined oligodendrocyte progenitor cell proliferation in the glial scar at 14 d after injury. No differences in the numbers of newly-generated Olig2+ cells were detected between VEH- and PUR-treated animals, although the total number of proliferating cells was decreased by PUR (Figure 7p).

In conclusion, our study demonstrated that the small-molecule SMO receptor agonist PUR is neuroprotective early after stroke, reduces inflammation and astrogliosis and promotes regeneration in the ischemic cortex at a later time point after ischemic insult.

## Discussion

Here we investigated the neuroprotective and regenerative properties of the Shh signaling agonist PUR and identified PUR as a potential candidate in ischemic stroke therapy. Our results demonstrated a previously unappreciated role of Shh signaling in neuroprotection via a novel tPA-dependent mechanism, indicating that reiteration of a developmental signaling pathway in the adult brain confers neuroprotective and regenerative effects on injury associated with stroke.

We found that at 9 h after permanent MCAO, neurons in the ipsilateral and contralateral hemispheres upregulated the expression of Shh, reinforcing previous reports of neuronal Shh expression in the normal brain and early after ischemia.^9,16,40^ Early after stroke, predominantly neurons but also astrocytes, oligodendrocytes, microglia and endothelial cells in the ischemic area upregulated Shh receptors expression. An increase of Gli1 in the ipsilateral cortex indicated the activation of the Shh pathway early after stroke.

The elevated level of Shh on the contralateral side early after MCAO was an interesting finding. Inflammation has been identified to trigger Shh signaling.^19^ In support, an increase of TNF*α*, IL1*β* and IL6 was detected in the ischemic cortex at 9 h after stroke. However, the lack of inflammation and an unchanged level of Gli1 mRNA on the contralateral side suggest that inflammation is not the only trigger of Shh signaling following stroke. Notably, the reduction of cerebral blood flow occurring after MCAO in the contralateral hemisphere^41^ could have a decisive role in the regulation of Shh signaling activation.

Although the endogenous Shh pathway is activated in response to stroke, its effect on cell survival remains unclear. In our treatment paradigm, inhibition of Smo receptors with CyP did not aggravate MCAO-induced cortical damage. Similar observation was reported after epidural application of CyP in an ischemia–reperfusion model.^10^ In contrast, intracerebral infusion of CyP immediately after permanent MCAO has been shown to be deleterious.^13^ In our study, a single i.v. administration of PUR at 6 h after stroke was neuroprotective and restored neurological deficits induced by ischemic injury. These findings point to the diverse role of endogenous Shh signaling at various time points after stroke and highlight the importance of defining a precise therapeutic time window for the clinical use of Shh agonists.

Administered at 6 h after stroke, PUR targeted apoptotic cells in the ischemic penumbra. The anti-apoptotic effect of PUR was associated with increased expression of tPA in penumbral neurons. tPA is a serine proteinase, which cleaves the plasminogen to active plasmin, an enzyme responsible for blood clot breakdown.^42^ In the brain, tPA is found in association with an endothelial cell–astrocyte complex, where it modulates cerebrovascular tone, functioning at low doses as vasodilator and at high doses as vasoconstrictor,^43^ and regulates the permeability of the BBB.^44^ Neuronal tPA is involved in synaptic plasticity.^45^ tPA-induced Ca^2+^ influx contributes to excitotoxicity, and tPA gene knockout has been shown to be neuroprotective.^46, 47, 48^ Several studies demonstrated a beneficial role of tPA in neuronal and oligodendrocyte survival.^49, 50, 51, 52^ Liot *et al.*^52^ showed that tPA protects cortical neurons against serum deprivation-induced apoptosis through a mechanism independent of tPA proteolytic activity or tPA receptors. Correa *et al.*^49^ demonstrated that tPA contains a domain of significant homology with epidermal growth factor and by binding to its receptors on oligodendrocytes inhibits apoptotic cell death after MCAO. Ischemic preconditioning increases tPA enzymatic activity in cortical and hippocampal neurons providing neuroprotection against a subsequent lethal ischemic insult.^50,51^ Consistent with previous observations,^53,54^ we found that the enzymatic activity of tPA was reduced in the ischemic cortex early after stroke. However, at 24 h, tPA enzymatic activity was increased in the PUR-treated group. The use of tPA in thrombolytic therapy of ischemic stroke is limited due to adverse complications, including hemorrhage.^1,55^ Notably, increase in tPA expression (at 9 h) and activity (at 24 h) by PUR was associated with neuroprotection and reduction of BBB permeability. Hence, PUR may be an alternative for delayed stroke therapy, potentially avoiding harmful side effects of tPA.

Concerning the safety of PUR as an agonist of potential oncogenic Shh signaling,^56^ no evidences of neoplastic transformation in peripheral or CNS tissues of PUR-treated animals were observed. In our treatment paradigm, PUR did not increase the level of Gli1 mRNA, associated with canonical Shh signaling, giving additional credit to the potential safe use of PUR in stroke therapy. Future studies on the possible involvement of non-canonical Shh signaling, acting through G*α*_i_,^57^ are warranted.

Astrocytes respond to CNS injury by proliferation and formation of the glial scar.^58^ The glial scar isolates damaged area from healthy environment, fills the lesion cavity, reduces immune cells infiltration and facilitates BBB repair.^59,60^ Glial scar and an inflammatory environment, however, restrict axonal sprouting and postinjury tissue regeneration.^58,60^ Reactive astrocytes express and self-respond to Shh that stimulates their proliferation and glial scar formation.^19,40^ PUR did not alter levels of Shh signaling molecules found at 7 d after insult. However, a reduction of glial scar and decreased immune cells' infiltration and inflammation were observed in the ischemic area after PUR treatment. Attenuation of astrogliosis by PUR could result from neuroprotection. PUR might also reduce the need for Shh signaling compensation by reactive astrocytes and thereby limit their proliferation in the injured CNS tissue. Additionally, PUR might directly affect the function of immune cells, leading to subsequent downregulation of glial scar formation.

Treatment with PUR promoted tissue regeneration after stroke. We found an increased number of neuroblasts in the ischemic area after PUR treatment. The majority of neuroblasts expressed growth-associated protein GAP43, indicating an outgrowth of neurites and the formation of new axonal projections.^37^ The origin of newly generated neurons found in the ischemic area is an interesting question. A recent study using a Cre-*LoxP* system to label SVZ-derived cells demonstrated that all new neurons found in the striatum after stroke were generated in SVZ and migrated along the blood vessels.^38^ In agreement, we observed chains of migrating neuroblasts distributed from the SVZ toward the ischemic area along the CC and striatum. Migrating cells were located in close vicinity to vascular endothelium. PUR increased neovascularization in the infarct area that supports neuroblasts migration and promotes tissue reconstruction.^36^

In summary, we have, for the first time, demonstrated the potential of the small-molecule Shh signaling pathway agonist PUR to protect cortical tissue and promote regeneration after ischemic stroke. These findings provide important insights into the role of Shh signaling in the adult CNS following ischemic stroke and may open new avenues in stroke therapy.

## Materials and Methods

### Animals

Experiments were carried out in accordance with the National Institutes of Health (NIH, Bethesda, MD, USA) guidelines for use of laboratory animals. All animal protocols were approved by the University of California at Davis Institutional Animal Care and Use Committee. All efforts were made to minimize the number of animals used and to ensure minimal suffering. C57BL/6 male mice 9–12-weeks old (The Jackson Laboratories, Sacramento, CA, USA) were used in this study.

### Focal cerebral ischemia and drug treatment

Focal ischemia was induced by permanent occlusion of the left middle cerebral artery (MCA) under isoflurane anesthesia as described previously.^61^ Briefly, a skin incision was made between the orbit and the ear. Under an operating microscope, the temporal muscle was divided to locate the distal course of the left MCA through the translucent skull. A small burr-hole craniotomy was performed with a microdrill (FST, Foster City, CA, USA) and the exposed MCA was coagulated using a bipolar pencil (Kirwan Surgical Products, Marshfield, MA, USA). Permanent occlusion was ensured by physical division. Animals were randomly divided into groups and received a single dose of 1, 5 and 15 mg/kg of PUR or VEH (PEG-400: ethanol: water (40 : 20 : 40) i.v. or i.p. at 6 h after MCAO. SHAM-operated animals were injected with VEH. CyP (10 mg/kg) was administered 40 min before PUR (Figure 1a). To label proliferating cells, 50 mg/kg BrdU (Roche, Indianapolis, IN, USA) in sterile 0.9% NaCl was administered i.p. daily from 4 to 7 d after MCAO (Figure 1a). For tissue collection, mice were anesthetized and perfused with cold phosphate-buffered saline (PBS). The ischemic area of the ipsilateral cortex and its contralateral counterpart was isolated and stored in liquid nitrogen for subsequent analysis.

### Behavioral assessment of neurological deficit

To assess somatosensory and motor function, an adhesive removal test was used as described previously.^62^ Animals were trained for 5 consecutive days prior to MCAO (Figure 1a). The time required to sense and remove adhesive was monitored at each trial. Standardized rotarod test was used to assess motor function of balance and coordination.^63^ Data were presented as the percentage to the time on the rotator recorded at the last day of training. All experiments were conducted in a blinded manner.

### Analysis of infarct volume

Infarct volume was examined at 48 h after MCAO using TTC and analyzed with correction for brain swelling as previously described.^64^

### Analysis of vascular permeability

BBB permeability was examined as previously described.^44^ Briefly, EB was injected i.v. at 9 h after stroke and animals were perfused with PBS 1 h later. Hemispheres were separated, weighed and homogenized in 400 *μ*l of N,N-dimethylformamide. Supernatants were collected after centrifugation at 25 000 × *g* for 45 min. EB extravasation was detected as the difference in absorbance at 620 nm and baseline absorbance (500–740 nm), relative to the wet weight of each hemisphere.

### RNA isolation and qPCR

To examine the mRNA expression levels of Shh signaling molecules and target genes, we performed qPCR analysis as described previously.^65^ Total RNA was isolated using RNeasy Lipid Tissue Mini Kit (Qiagen, Valencia, CA, USA) and reverse-transcribed to cDNA using MultiscribeTM reverse transcriptase (Applied Biosystems, Foster City, CA, USA). Subsequent qPCR reactions were performed on a Roche Lightcycler 480 using SYBR Green Master Mix (Roche Applied Science, Indianapolis, IN, USA) or validated TaqMan gene expression assay (Applied Biosystems). Relative quantification of the fold-change was performed by applying the 2^−ΔΔ^*Ct* method and comparing *Cp* values (calculated by a second-derivative maximum) of individual mice to sham-operated control mice.^66^ Primer details are included in Supplementary Materials and Methods.

### Western blotting analysis

We isolated proteins from cortical tissues using the NE-PER nuclear and cytoplasmic extraction kit (ThermoScientific, Waltham, MA, USA). Proteins were separated on SDS-PAGE and electrotransferred onto PVDF membrane (Bio-Rad, Hercules, CA, USA). Gels were run under the same conditions for all experimental groups. Blots were probed with specific antibodies followed by incubation with horseradish peroxidase-conjugated secondary antibody and developed using ECL reagent (Amersham Biosciences, Piscataway, NJ, USA). *β*-Actin or Lamin B1 was detected for each membrane as the cytoplasmic or nuclear protein loading control, respectively. The intensity of the bands was analyzed using Image J (NIH). Data are presented as the relative ratio to the loading control. For data display, representative bands cropped from the same blot are indicated by a vertical separation line. Detailed information about primary antibodies is included in Supplementary Materials and Methods.

### Direct casein zymography for tPA activity

tPA activity was assessed by direct zymography as described elsewhere.^53,67^ Briefly, proteins of cortical tissue lysates were separated by electrophoresis on resolving gel (11% acrylamide) containing 1 mg/ml casein and 13 *μ*g/ml plasminogen (rPeptide, Bogart, GA, USA) as substrates for plasmin and PA, respectively. The gel was washed and incubated for 4 h at 37 °C in 0.1 M Tris buffer to enable caseinolysis. Following Coomassie Brilliant Blue R-250 staining, tPA activity was visualized as light bands resulting from casein degradation on the darkly stained casein background. To avoid loading variations, independent duplicate samples were analyzed by western blotting for *β*-actin.

### Immunohistochemistry

Brain tissues were collected and prepared for staining as described earlier.^65^ Nonspecific binding was blocked using 10% goat serum in 0.1% Triton-X in PBS. Sections were incubated overnight at 4 °C with primary antibody, and specific binding was detected with secondary antibody Alexa Fluor 488 or 555 (Invitrogen, Carlsbad, CA, USA). Primary antibody was omitted in negative controls. Images were acquired on a Nikon Eclipse TE 2000-E confocal microscope using a D-Eclipse C1si camera (Nikon Instruments Inc., Melville, NY, USA). Brightness was adjusted for some images to improve their presentational quality without compromising data accuracy. For vascular density and neovascularization analysis, six sections per animal with an interval of 210 *μ*m were stained for BrdU and CD31. Animals from three independent experiments were included in the analysis. Images were obtained in two 300 × 300 *μ*m^2^ areas placed in the penumbra or the infarct zone. Vascular density was calculated and presented as the percentage of the area occupied by CD31+ vessels over the total area using Adobe Photoshop CS3 (San Jose, CA, USA). BrdU/CD31+ cells were counted for neovascularization analysis and presented as the mean number of cells per mm^2^. Analysis was performed by blinded investigators. Detailed information on primary antibodies, method of glial scar analysis and cell quantification is included in Supplementary Materials and Methods and Supplementary Figures.

### TUNEL staining

TUNEL assay was performed on 14-*μ*m frozen tissue sections using *In Situ* Cell Death Detection Kit (Roche) following the manufacturer's instructions. To label neurons, sections were counterstained with NeuN.

### Statistical analysis

All statistical analyses were performed using GraphPad Prism 5.0 (GraphPad Software Inc., San Diego, CA USA). Statistical significance of the differences between means was evaluated using one-way ANOVA followed by Turkey's *post hoc* test for multiple comparisons. Single comparisons were analyzed by the Student's *t*-test. All data were represented as mean±S.E.M. Probability values <0.05 were considered statistically significant.

## Abbreviations

BBB: blood–brain barrier
CC: corpus callosum
CNS: central nervous system
CyP: cyclopamine
EB: Evans Blue
GFAP: glial fibrillary acidic protein
i.p.: intraperitoneal
i.v.: intravenous
MCAO: middle cerebral artery occlusion
Ptch1: Patched 1
PUR: purmorphamine
Shh: sonic hedgehog
SMO: Smoothened
SVZ: subventricular zone
tPA: tissue-type plasminogen activator
TTC: 2,3,5-triphenyltetrazolium chloride
VEH: vehicle

## References

[bib1] 1Shamy MC, Jaigobin CS. The complexities of acute stroke decision-making: a survey of neurologists. Neurology 2013; 81: 1130–1133. 23946306 10.1212/WNL.0b013e3182a55ec7

[bib2] 2Traiffort E, Angot E, Ruat M. Sonic Hedgehog signaling in the mammalian brain. J Neurochem 2010; 113: 576–590. 20218977 10.1111/j.1471-4159.2010.06642.x

[bib3] 3Charytoniuk D, Porcel B, Rodriguez Gomez J, Faure H, Ruat M, Traiffort E. Sonic Hedgehog signalling in the developing and adult brain. J Physiol Paris 2002; 96: 9–16. 11755778 10.1016/s0928-4257(01)00075-4

[bib4] 4Hynes M, Porter JA, Chiang C, Chang D, Tessier-Lavigne M, Beachy PA et al. Induction of midbrain dopaminergic neurons by Sonic hedgehog. Neuron 1995; 15: 35–44. 7619528 10.1016/0896-6273(95)90062-4

[bib5] 5Lu QR, Yuk D, Alberta JA, Zhu Z, Pawlitzky I, Chan J et al. Sonic hedgehog--regulated oligodendrocyte lineage genes encoding bHLH proteins in the mammalian central nervous system. Neuron 2000; 25: 317–329. 10719888 10.1016/s0896-6273(00)80897-1

[bib6] 6Dahmane N, Ruiz i Altaba A. Sonic hedgehog regulates the growth and patterning of the cerebellum. Development 1999; 126: 3089–3100. 10375501 10.1242/dev.126.14.3089

[bib7] 7Ihrie RA, Shah JK, Harwell CC, Levine JH, Guinto CD, Lezameta M et al. Persistent sonic hedgehog signaling in adult brain determines neural stem cell positional identity. Neuron 2011; 71: 250–262. 21791285 10.1016/j.neuron.2011.05.018 PMC3346180

[bib8] 8Han YG, Spassky N, Romaguera-Ros M, Garcia-Verdugo JM, Aguilar A, Schneider-Maunoury S et al. Hedgehog signaling and primary cilia are required for the formation of adult neural stem cells. Nat Neurosci 2008; 11: 277–284. 18297065 10.1038/nn2059

[bib9] 9Garcia AD, Petrova R, Eng L, Joyner AL. Sonic hedgehog regulates discrete populations of astrocytes in the adult mouse forebrain. J Neurosci 2010; 30: 13597–13608. 20943901 10.1523/JNEUROSCI.0830-10.2010 PMC2966838

[bib10] 10Huang SS, Cheng H, Tang CM, Nien MW, Huang YS, Lee IH et al. Anti-oxidative, anti-apoptotic, and pro-angiogenic effects mediate functional improvement by sonic hedgehog against focal cerebral ischemia in rats. Exp Neurol 2013; 247: 680–688. 23499832 10.1016/j.expneurol.2013.03.004

[bib11] 11Alvarez JI, Dodelet-Devillers A, Kebir H, Ifergan I, Fabre PJ, Terouz S et al. The Hedgehog pathway promotes blood-brain barrier integrity and CNS immune quiescence. Science 2011; 334: 1727–1731. 22144466 10.1126/science.1206936

[bib12] 12Jenkins D. Hedgehog signalling: emerging evidence for non-canonical pathways. Cell Signal 2009; 21: 1023–1034. 19399989 10.1016/j.cellsig.2009.01.033

[bib13] 13Ji H, Miao J, Zhang X, Du Y, Liu H, Li S et al. Inhibition of sonic hedgehog signaling aggravates brain damage associated with the down-regulation of Gli1, Ptch1 and SOD1 expression in acute ischemic stroke. Neurosci Lett 2012; 506: 1–6. 22133807 10.1016/j.neulet.2011.11.027

[bib14] 14Bambakidis NC, Petrullis M, Kui X, Rothstein B, Karampelas I, Kuang Y et al. Improvement of neurological recovery and stimulation of neural progenitor cell proliferation by intrathecal administration of Sonic hedgehog. J Neurosurg 2012; 116: 1114–1120. 22324418 10.3171/2012.1.JNS111285

[bib15] 15Dellovade T, Romer JT, Curran T, Rubin LL. The hedgehog pathway and neurological disorders. Annu Rev Neurosci 2006; 29: 539–563. 16776596 10.1146/annurev.neuro.29.051605.112858

[bib16] 16Sims JR, Lee SW, Topalkara K, Qiu J, Xu J, Zhou Z et al. Sonic hedgehog regulates ischemia/hypoxia-induced neural progenitor proliferation. Stroke 2009; 40: 3618–3626. 19762700 10.1161/STROKEAHA.109.561951 PMC2869495

[bib17] 17Zhang J, Li Y, Zhang ZG, Lu M, Borneman J, Buller B et al. Bone marrow stromal cells increase oligodendrogenesis after stroke. J Cereb Blood Flow Metab 2009; 29: 1166–1174. 19384336 10.1038/jcbfm.2009.41 PMC2849641

[bib18] 18Xia YP, He QW, Li YN, Chen SC, Huang M, Wang Y et al. Recombinant human sonic hedgehog protein regulates the expression of ZO-1 and occludin by activating angiopoietin-1 in stroke damage. PLoS One 2013; 8: e68891. 23894369 10.1371/journal.pone.0068891 PMC3720889

[bib19] 19Amankulor NM, Hambardzumyan D, Pyonteck SM, Becher OJ, Joyce JA, Holland EC. Sonic hedgehog pathway activation is induced by acute brain injury and regulated by injury-related inflammation. J Neurosci 2009; 29: 10299–10308. 19692604 10.1523/JNEUROSCI.2500-09.2009 PMC3849831

[bib20] 20Tsuboi K, Shults CW. Intrastriatal injection of sonic hedgehog reduces behavioral impairment in a rat model of Parkinson's disease. Exp Neurol 2002; 173: 95–104. 11771942 10.1006/exnr.2001.7825

[bib21] 21Dass B, Iravani MM, Huang C, Barsoum J, Engber TM, Galdes A et al. Sonic hedgehog delivered by an adeno-associated virus protects dopaminergic neurones against 6-OHDA toxicity in the rat. J Neural Transm 2005; 112: 763–778. 15586304 10.1007/s00702-004-0227-7

[bib22] 22Reilly JO, Karavanova ID, Williams KP, Mahanthappa NK, Allendoerfer KL. Cooperative effects of Sonic Hedgehog and NGF on basal forebrain cholinergic neurons. Mol Cell Neurosci 2002; 19: 88–96. 11817900 10.1006/mcne.2001.1063

[bib23] 23Seifert T, Bauer J, Weissert R, Fazekas F, Storch MK. Differential expression of sonic hedgehog immunoreactivity during lesion evolution in autoimmune encephalomyelitis. J Neuropathol Exp Neurol 2005; 64: 404–411. 15892298 10.1093/jnen/64.5.404

[bib24] 24Franco PG, Silvestroff L, Soto EF, Pasquini JM. Thyroid hormones promote differentiation of oligodendrocyte progenitor cells and improve remyelination after cuprizone-induced demyelination. Exp Neurol 2008; 212: 458–467. 18572165 10.1016/j.expneurol.2008.04.039

[bib25] 25Bambakidis NC, Wang X, Lukas RJ, Spetzler RF, Sonntag VK, Preul MC. Intravenous hedgehog agonist induces proliferation of neural and oligodendrocyte precursors in rodent spinal cord injury. Neurosurgery 2010; 67: 1709–1715 discussion 1715. 21107202 10.1227/NEU.0b013e3181f9b0a5

[bib26] 26Bambakidis NC, Horn EM, Nakaji P, Theodore N, Bless E, Dellovade T et al. Endogenous stem cell proliferation induced by intravenous hedgehog agonist administration after contusion in the adult rat spinal cord. J Neurosurg Spine 2009; 10: 171–176. 19278333 10.3171/2008.10.SPI08231

[bib27] 27Peterson R, Turnbull J. Sonic hedgehog is cytoprotective against oxidative challenge in a cellular model of amyotrophic lateral sclerosis. J Mol Neurosci 2011; 47: 31–41. 21979788 10.1007/s12031-011-9660-x

[bib28] 28Heine VM, Griveau A, Chapin C, Ballard PL, Chen JK, Rowitch DH. A small-molecule smoothened agonist prevents glucocorticoid-induced neonatal cerebellar injury. Sci Transl Med 2011; 3: 105ra104. 10.1126/scitranslmed.3002731 PMC3694585 22013124

[bib29] 29Sinha S, Chen JK. Purmorphamine activates the Hedgehog pathway by targeting Smoothened. Nat Chem Biol 2006; 2: 29–30. 16408088 10.1038/nchembio753

[bib30] 30Wu X, Ding S, Ding Q, Gray NS, Schultz PG. A small molecule with osteogenesis-inducing activity in multipotent mesenchymal progenitor cells. J Am Chem Soc 2002; 124: 14520–14521. 12465946 10.1021/ja0283908

[bib31] 31Lipton P. Ischemic cell death in brain neurons. Physiol Rev 1999; 79: 1431–1568. 10508238 10.1152/physrev.1999.79.4.1431

[bib32] 32Chechneva O, Dinkel K, Cavaliere F, Martinez-Sanchez M, Reymann KG. Anti-inflammatory treatment in oxygen-glucose-deprived hippocampal slice cultures is neuroprotective and associated with reduced cell proliferation and intact neurogenesis. Neurobiol Dis 2006; 23: 247–259. 16733089 10.1016/j.nbd.2006.02.015

[bib33] 33Nakashima H, Nakamura M, Yamaguchi H, Yamanaka N, Akiyoshi T, Koga K et al. Nuclear factor-kappaB contributes to hedgehog signaling pathway activation through sonic hedgehog induction in pancreatic cancer. Cancer Res 2006; 66: 7041–7049. 16849549 10.1158/0008-5472.CAN-05-4588

[bib34] 34Dai RL, Zhu SY, Xia YP, Mao L, Mei YW, Yao YF et al. Sonic hedgehog protects cortical neurons against oxidative stress. Neurochem Res 2011; 36: 67–75. 20848190 10.1007/s11064-010-0264-6

[bib35] 35Su EJ, Fredriksson L, Geyer M, Folestad E, Cale J, Andrae J et al. Activation of PDGF-CC by tissue plasminogen activator impairs blood-brain barrier integrity during ischemic stroke. Nat Med 2008; 14: 731–737. 18568034 10.1038/nm1787 PMC2811427

[bib36] 36Ohab JJ, Fleming S, Blesch A, Carmichael ST. A neurovascular niche for neurogenesis after stroke. J Neurosci 2006; 26: 13007–13016. 17167090 10.1523/JNEUROSCI.4323-06.2006 PMC6674957

[bib37] 37Strittmatter SM, Igarashi M, Fishman MC. GAP-43 amino terminal peptides modulate growth cone morphology and neurite outgrowth. J Neurosci 1994; 14: 5503–5513. 8083750 10.1523/JNEUROSCI.14-09-05503.1994 PMC6577098

[bib38] 38Yamashita T, Ninomiya M, Hernandez Acosta P, Garcia-Verdugo JM, Sunabori T, Sakaguchi M et al. Subventricular zone-derived neuroblasts migrate and differentiate into mature neurons in the post-stroke adult striatum. J Neurosci 2006; 26: 6627–6636. 16775151 10.1523/JNEUROSCI.0149-06.2006 PMC6674034

[bib39] 39Janz R, Sudhof TC, Hammer RE, Unni V, Siegelbaum SA, Bolshakov VY. Essential roles in synaptic plasticity for synaptogyrin I and synaptophysin I. Neuron 1999; 24: 687–700. 10595519 10.1016/s0896-6273(00)81122-8

[bib40] 40Sirko S, Behrendt G, Johansson PA, Tripathi P, Costa M, Bek S et al. Reactive glia in the injured brain acquire stem cell properties in response to sonic hedgehog glia. Cell Stem Cell 2013; 12: 426–439. 23561443 10.1016/j.stem.2013.01.019

[bib41] 41Laing RJ, Jakubowski J, Laing RW. Middle cerebral artery occlusion without craniectomy in rats. Which method works best? Stroke 1993; 24: 294–297 discussion 297–298. 8421831 10.1161/01.str.24.2.294

[bib42] 42Collen D, Lijnen HR. Basic and clinical aspects of fibrinolysis and thrombolysis. Blood 1991; 78: 3114–3124. 1742478

[bib43] 43Nassar T, Akkawi S, Shina A, Haj-Yehia A, Bdeir K, Tarshis M et al. *In vitro* and *in vivo* effects of tPA and PAI-1 on blood vessel tone. Blood 2004; 103: 897–902. 14512309 10.1182/blood-2003-05-1685

[bib44] 44Yepes M, Sandkvist M, Moore EG, Bugge TH, Strickland DK, Lawrence DA. Tissue-type plasminogen activator induces opening of the blood-brain barrier via the LDL receptor-related protein. J Clin Invest 2003; 112: 1533–1540. 14617754 10.1172/JCI19212 PMC259131

[bib45] 45Qian Z, Gilbert ME, Colicos MA, Kandel ER, Kuhl D. Tissue-plasminogen activator is induced as an immediate-early gene during seizure, kindling and long-term potentiation. Nature 1993; 361: 453–457. 8429885 10.1038/361453a0

[bib46] 46Nicole O, Docagne F, Ali C, Margaill I, Carmeliet P, MacKenzie ET et al. The proteolytic activity of tissue-plasminogen activator enhances NMDA receptor-mediated signaling. Nat Med 2001; 7: 59–64. 11135617 10.1038/83358

[bib47] 47Yepes M, Sandkvist M, Wong MK, Coleman TA, Smith E, Cohan SL et al. Neuroserpin reduces cerebral infarct volume and protects neurons from ischemia-induced apoptosis. Blood 2000; 96: 569–576. 10887120

[bib48] 48Tsirka SE, Gualandris A, Amaral DG, Strickland S. Excitotoxin-induced neuronal degeneration and seizure are mediated by tissue plasminogen activator. Nature 1995; 377: 340–344. 7566088 10.1038/377340a0

[bib49] 49Correa F, Gauberti M, Parcq J, Macrez R, Hommet Y, Obiang P et al. Tissue plasminogen activator prevents white matter damage following stroke. J Exp Med 2011; 208: 1229–1242. 21576385 10.1084/jem.20101880 PMC3173251

[bib50] 50Echeverry R, Wu J, Haile WB, Guzman J, Yepes M. Tissue-type plasminogen activator is a neuroprotectant in the mouse hippocampus. J Clin Invest 2010; 120: 2194–2205. 20440070 10.1172/JCI41722 PMC2877952

[bib51] 51Haile WB, Wu J, Echeverry R, Wu F, An J, Yepes M. Tissue-type plasminogen activator has a neuroprotective effect in the ischemic brain mediated by neuronal TNF-alpha. J Cereb Blood Flow Metab 2012; 32: 57–69. 21792242 10.1038/jcbfm.2011.106 PMC3323291

[bib52] 52Liot G, Roussel BD, Lebeurrier N, Benchenane K, Lopez-Atalaya JP, Vivien D et al. Tissue-type plasminogen activator rescues neurones from serum deprivation-induced apoptosis through a mechanism independent of its proteolytic activity. J Neurochem 2006; 98: 1458–1464. 16800849 10.1111/j.1471-4159.2006.03982.x

[bib53] 53Ahn MY, Zhang ZG, Tsang W, Chopp M. Endogenous plasminogen activator expression after embolic focal cerebral ischemia in mice. Brain Res 1999; 837: 169–176. 10433999 10.1016/s0006-8993(99)01645-5

[bib54] 54Rosenberg GA, Navratil M, Barone F, Feuerstein G. Proteolytic cascade enzymes increase in focal cerebral ischemia in rat. J Cereb Blood Flow Metab 1996; 16: 360–366. 8621740 10.1097/00004647-199605000-00002

[bib55] 55Mohr JP. Thrombolytic therapy for ischemic stroke: from clinical trials to clinical practice. JAMA 2000; 283: 1189–1191. 10703782 10.1001/jama.283.9.1189

[bib56] 56Hui CC, Angers S. Gli proteins in development and disease. Annu Rev Cell Dev Biol 2011; 27: 513–537. 21801010 10.1146/annurev-cellbio-092910-154048

[bib57] 57Ogden SK, Fei DL, Schilling NS, Ahmed YF, Hwa J, Robbins DJ. G protein Galphai functions immediately downstream of Smoothened in Hedgehog signalling. Nature 2008; 456: 967–970. 18987629 10.1038/nature07459 PMC2744466

[bib58] 58Sofroniew MV. Molecular dissection of reactive astrogliosis and glial scar formation. Trends Neurosci 2009; 32: 638–647. 19782411 10.1016/j.tins.2009.08.002 PMC2787735

[bib59] 59Bush TG, Puvanachandra N, Horner CH, Polito A, Ostenfeld T, Svendsen CN et al. Leukocyte infiltration, neuronal degeneration, and neurite outgrowth after ablation of scar-forming, reactive astrocytes in adult transgenic mice. Neuron 1999; 23: 297–308. 10399936 10.1016/s0896-6273(00)80781-3

[bib60] 60Li L, Lundkvist A, Andersson D, Wilhelmsson U, Nagai N, Pardo AC et al. Protective role of reactive astrocytes in brain ischemia. J Cereb Blood Flow Metab 2008; 28: 468–481. 17726492 10.1038/sj.jcbfm.9600546

[bib61] 61Koistinaho M, Malm TM, Kettunen MI, Goldsteins G, Starckx S, Kauppinen RA et al. Minocycline protects against permanent cerebral ischemia in wild type but not in matrix metalloprotease-9-deficient mice. J Cereb Blood Flow Metab 2005; 25: 460–467. 15674236 10.1038/sj.jcbfm.9600040

[bib62] 62Bouet V, Boulouard M, Toutain J, Divoux D, Bernaudin M, Schumann-Bard P et al. The adhesive removal test: a sensitive method to assess sensorimotor deficits in mice. Nat Protoc 2009; 4: 1560–1564. 19798088 10.1038/nprot.2009.125

[bib63] 63Hunter AJ, Hatcher J, Virley D, Nelson P, Irving E, Hadingham SJ et al. Functional assessments in mice and rats after focal stroke. Neuropharmacology 2000; 39: 806–816. 10699446 10.1016/s0028-3908(99)00262-2

[bib64] 64Swanson RA, Morton MT, Tsao-Wu G, Savalos RA, Davidson C, Sharp FR. A semiautomated method for measuring brain infarct volume. J Cereb Blood Flow Metab 1990; 10: 290–293. 1689322 10.1038/jcbfm.1990.47

[bib65] 65Chechneva OV, Mayrhofer F, Daugherty DJ, Pleasure DE, Hong JS, Deng W. Low dose dextromethorphan attenuates moderate experimental autoimmune encephalomyelitis by inhibiting NOX2 and reducing peripheral immune cells infiltration in the spinal cord. Neurobiol Dis 2011; 44: 63–72. 21704706 10.1016/j.nbd.2011.06.004 PMC3153572

[bib66] 66Livak KJ, Schmittgen TD. Analysis of relative gene expression data using real-time quantitative PCR and the 2(-Delta Delta C(T)) method. Methods 2001; 25: 402–408. 11846609 10.1006/meth.2001.1262

[bib67] 67Heussen C, Dowdle EB. Electrophoretic analysis of plasminogen activators in polyacrylamide gels containing sodium dodecyl sulfate and copolymerized substrates. Anal Biochem 1980; 102: 196–202. 7188842 10.1016/0003-2697(80)90338-3

