# Supplementary figures and images for: A Smoothened receptor agonist is neuroprotective and promotes regeneration after ischemic brain injury
Source: Cell Death Dis. 2014 Oct 23;5(10):e1481–. doi: 10.1038/cddis.2014.446 (PMC4649529; doi:10.1038/cddis.2014.446)

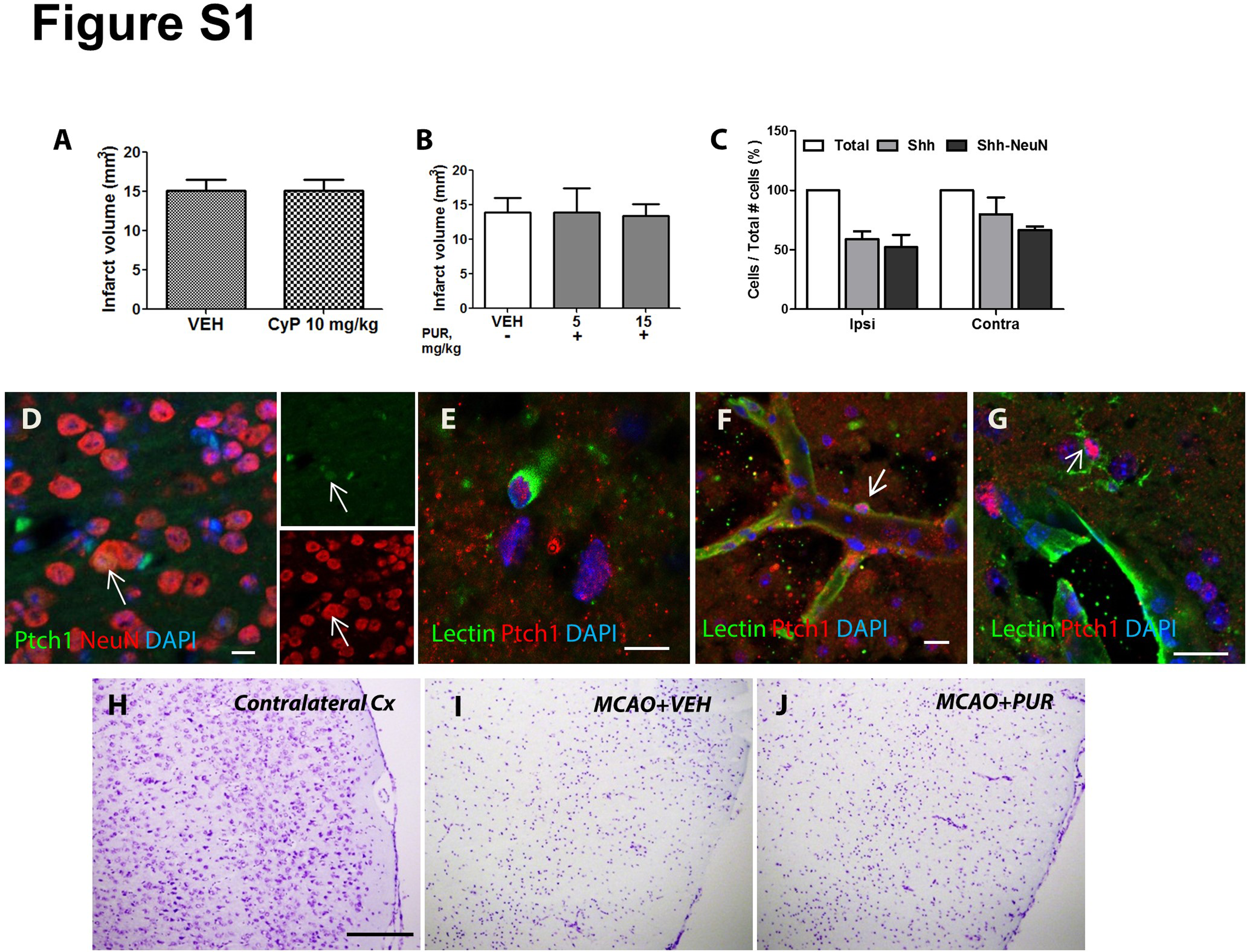

Supplement: Supplementary Figure S1 [file cddis2014446x1.tif]

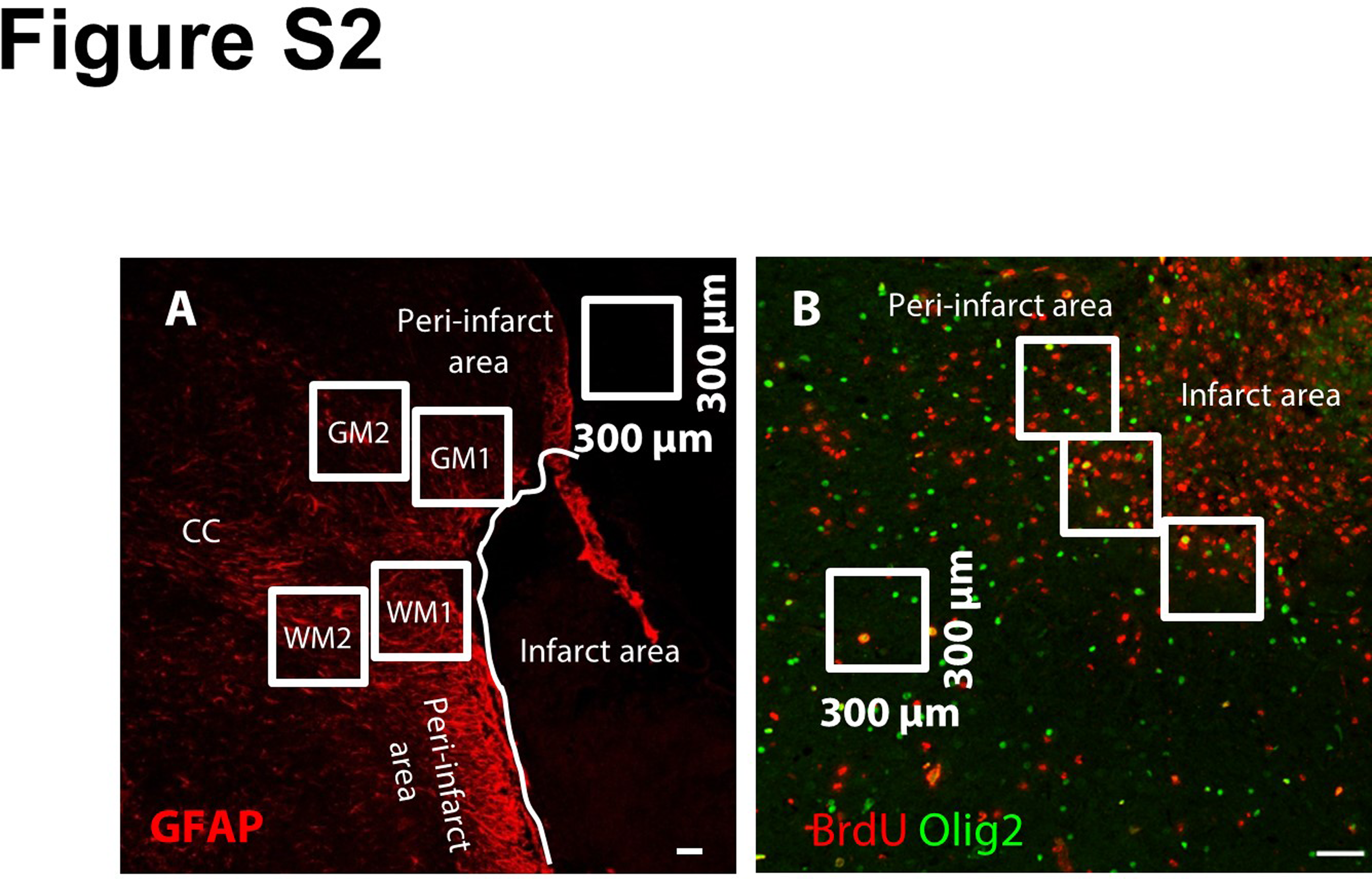

Supplement: Supplementary Figure S2 [file cddis2014446x2.tif]
